# The substantial economic burden of rotator cuff pathology: A nationwide cost‐of‐illness study

**DOI:** 10.1002/ksa.70411

**Published:** 2026-05-22

**Authors:** Umile Giuseppe Longo, Martina Marino, Vincenzo Candela, Silvia D'Amario, Daniela Martini, Andrea Bucciarelli, Ranieri Poli, Pieter D'Hooghe, Alessandro de Sire, Michele Mercurio, Arianna Carnevale

**Affiliations:** ^1^ Fondazione Policlinico Universitario Campus Bio‐Medico Rome Italy; ^2^ Research Unit of Orthopaedic and Trauma Surgery, Department of Medicine and Surgery Università Campus Bio‐Medico di Roma Rome Italy; ^3^ Italian Workers' Compensation Authority (INAIL) Rome Italy; ^4^ Ministero della salute, Dipartimento della salute umana, della salute animale e dell'ecosistema (One Health) e dei rapporti internazionali Ufficio 4 Rapporti internazionali Rome Italy; ^5^ Orthopaedic Surgeon and Assistant Chief of Surgery for Research, Department of Orthopaedic Surgery and Sportsmedicine Aspetar Hospital Doha Qatar; ^6^ Physical Medicine and Rehabilitation Unit, Department of Medical and Surgical Sciences University of Catanzaro “Magna Graecia” Catanzaro Italy; ^7^ Research Center on Musculoskeletal Health, MusculoSkeletalHealth@UMG University of Catanzaro “Magna Graecia” Catanzaro Italy; ^8^ Magna Graecia University & R. Dulbecco University Hospital of Catanzaro Catanzaro Italy

**Keywords:** degenerative disease, economic burden, healthcare expenditure, occupational disease, rotator cuff disease

## Abstract

**Purpose:**

The purpose of this study is to evaluate the nationwide economic burden of rotator cuff (RC) pathology by quantifying expenditures related to outpatient consultations including out‐of‐pocket expenses, hospitalisations and occupational insurance claims, within a national healthcare system.

**Methods:**

To estimate outpatient costs and to analyse hospital admissions with a primary or secondary diagnosis related to RC pathology, data from 2017 to 2023 were retrieved from the Italian Ministry of Health outpatient specialist care database and SDO database, respectively. Data from INAIL's statistical archives was extracted focusing exclusively on recognised cases of occupational disease between 2019 and 2023. Descriptive statistics were used to summarise annual service volumes, costs and data trends.

**Results:**

Outpatient service costs for a standard conservative treatment in the year 2023 amounted to approximately €1.7 billion. Theoretical remuneration for hospital discharges with primary diagnoses related to shoulder and RC pathologies revealed an annual expenditure for the year 2023 of €111 million and a total expenditure of €739 million from 2017 to 2023. These costs, based on diagnosis‐related group (DRG) reimbursements, explicitly include all surgical expenses. Based on INAIL data, the average cost per occupational shoulder disease case for 2024 was estimated at approximately €25,700 per worker, and over 5 years, several €100 million were spent.

**Conclusion:**

The annual economic burden of RC pathology in Italy exceeds €2 billion yearly, coming from private and public spending related to conservative outpatient treatment services, hospital admissions and expenditure for work‐related injuries. This figure provides a lower‐bound estimate of the total economic burden, and future cost‐analysis would benefit from consideration of indirect and physiotherapy‐related costs.

**Level of Evidence:**

Level III.

**Abbreviations**
CUTU: unified commission on the integrated tariff
DRG: diagnosis‐related group
GP: general practitioner
ICD‐10‐CM: International Classification of Diseases, 10th Revision, Clinical Modification Codes
ICD‐9‐CM: International Classification of Diseases, 9th Revision, Clinical Modification Codes
INAIL: Italian National Institute for Insurance against Accidents at Work
IRB: institutional review board
OOP: out‐of‐pocket
RC: rotator cuff
RIAP: Italian Arthroplasty Registry
SDO: Italian national outpatient visits records, national hospital discharge records
SSN: Italian National Health Service

## INTRODUCTION

Rotator cuff (RC) pathology represents the most common cause of shoulder pain and disability, affecting an estimated 10%–30% of the general adult population [1, 3, 4, 6, 12], with both incidence and prevalence increasing with age [1, 3, 4, 6, 12].

To estimate the prevalence of RC pathology and its surgical treatment, Italian nationwide registry studies [12] were combined with international prevalence data [16, 27]. Prevalence of RC pathology was reported to be 20.7% of the general population [27], of which only 34.2% was classified as symptomatic. This can be applied in proportion to the latest data published by the Italian National Institute of Statistics, which reports a population of approximately 58.9 million as of January 2025 [16].

Besides the large burden on patient quality of life [10], management of RC pathology is lengthy and costly [5, 6, 8, 11]. Its multifactorial pathogenesis involves a prominent intrinsic pathway characterised by chronic degenerative changes, including age‐related tendon degradation, microvascular impairment, and a diminished capacity for tissue repair [28, 29]. These alterations are often accompanied by persistent inflammation and fatty infiltration, further compromising tendon integrity [28, 29]. Given this degenerative nature, conservative management remains the gold‐standard first‐line strategy for treatment. It typically begins with the patient's general practitioner (GP), who oversees initial screening and a variety of nonoperative interventions [9]. Diagnosis alone often requires several imaging techniques [19], which may be repeated over time, while typical treatment regimens usually begin conservatively, and then may escalate to surgery [11]. The mean time between the onset of symptoms and surgical intervention is between 2.5 and 5 years [4, 7, 9, 19].

Overall, RC pathology results in an extensive use of resources for both the Italian National Health Service (SSN) and patients that sustain out‐of‐pocket (OOP) costs. Additionally, despite its prevalence, studies evaluating the nationwide cost‐of‐illness of this pathology are few and far between [14, 15]. An in‐depth understanding of its economic burden, which remains currently unavailable, may lead to a better understanding of where SNN and OOP expenditures lie and can aid in finding areas to both optimise costs in the treatment of RC pathology and improve the quality of treatment algorithms.

Therefore, the purpose of this study is to evaluate the nationwide economic burden of RC pathology by quantifying expenditures related to outpatient consultations including OOP expenses, hospitalisations and occupational insurance claims, within a national healthcare system.

## MATERIALS AND METHODS

The present study was designed as a nationwide, retrospective cost‐of‐illness study. Data were systematically retrieved from three primary national registries to capture a comprehensive view of healthcare utilisation and expenditure: the national hospital discharge records (SDO) and the national outpatient visits records, both managed by the Ministry of Health, and occupational injury records managed by the National Institute for Insurance against Accidents at Work (INAIL).

As this study utilised fully anonymised, aggregated administrative data provided by national authorities, it was conducted in accordance with national privacy legislation and did not require formal Institutional Review Board (IRB) or ethics committee approval.

### Outpatient cost analysis

To estimate outpatient costs, data were retrieved from the Italian Ministry of Health through a formal extraction request. The information included specific diagnostic and therapeutic procedures from the outpatient specialist care database over a 7‐year period (2017–2023).

The analysis was based on data extracted from the administrative flow of outpatient specialist care services, as collected through the national health information system. This data source includes routinely collected healthcare records submitted by providers for reimbursement purposes, encompassing procedure codes, dates of service, clinical settings and cost of service including both the cost borne by patients as co‐payments OOP and the cost covered by the SSN.

**Figure 1:**
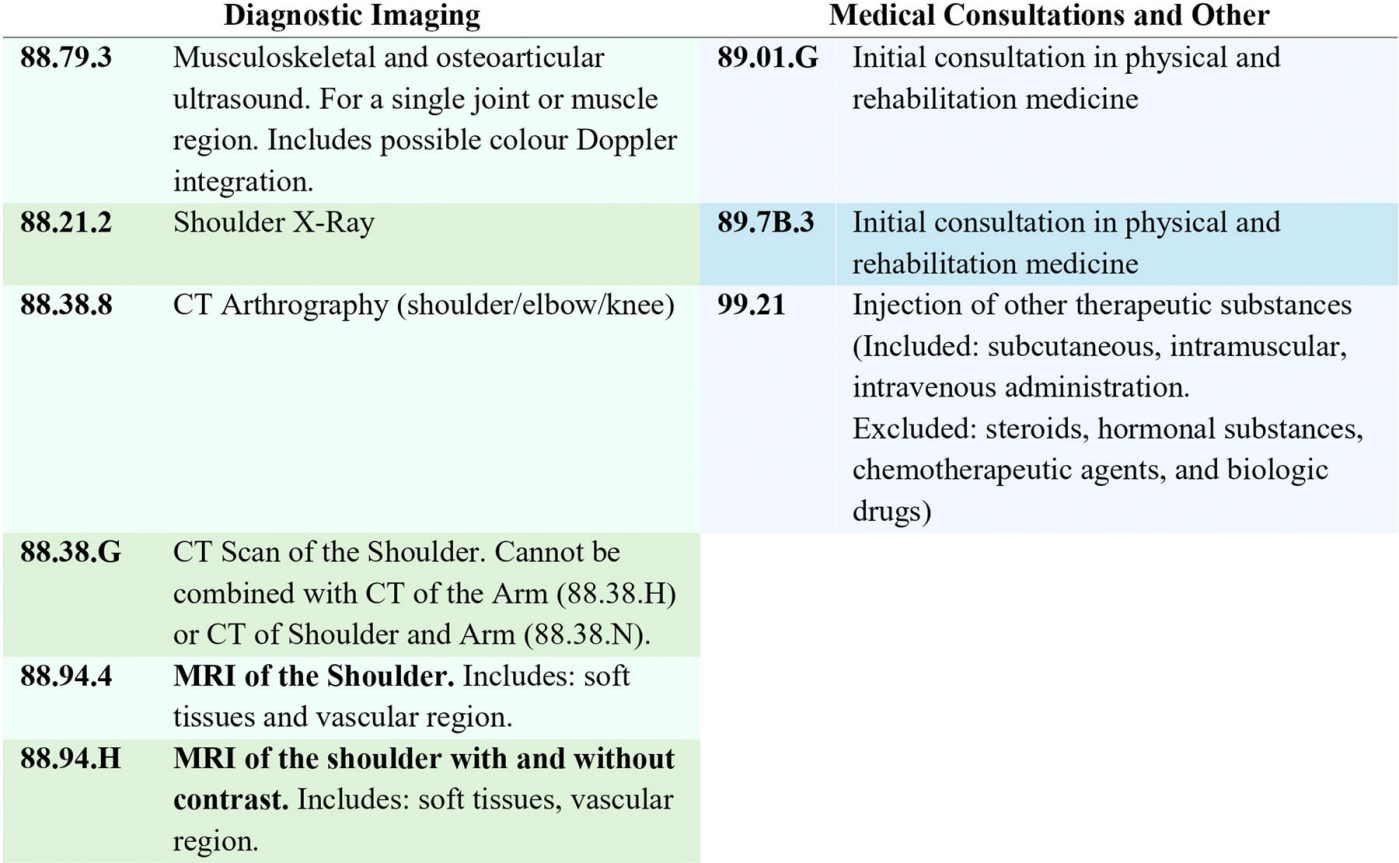
Requested extraction codes for all outpatient services related to RC pathology. CT, computed tomography; MRI, magnetic resonance imaging; RC, rotator cuff.

The extraction focused on identifying all outpatient services related to the following clinical areas: diagnostic imaging including conventional radiology, ultrasound, computed tomography (CT) and magnetic resonance imaging (MRI); instrumental examinations including functional diagnostic assessments; orthopaedic and rehabilitative consultations including first‐time and follow‐up visits performed by orthopaedic and physical and rehabilitation medicine specialists. Figure 1 shows the extraction codes of each service.

All relevant procedure codes were identified and selected from the official outpatient service nomenclature, matching the codes found in the administrative flow. Services were categorised by type and clinical objective, and codes were mapped to diagnostic and therapeutic pathways relevant to musculoskeletal disorders, with a focus on shoulder‐related conditions.

If extracted data did not consistently link each procedure code to a corresponding diagnosis code, an estimate of the proportion of these procedures potentially attributable to RC pathology was made. The estimated attribution percentages were based on epidemiological and clinical evidence from Italian and European data sources for ensuring robustness and conservativeness of the analysis.

### Hospital discharge records cost analysis

An extraction from the SDO database of the Italian Ministry of Health was performed to analyse hospital admissions with a primary or secondary diagnosis related to RC pathology.

**Figure 2:**
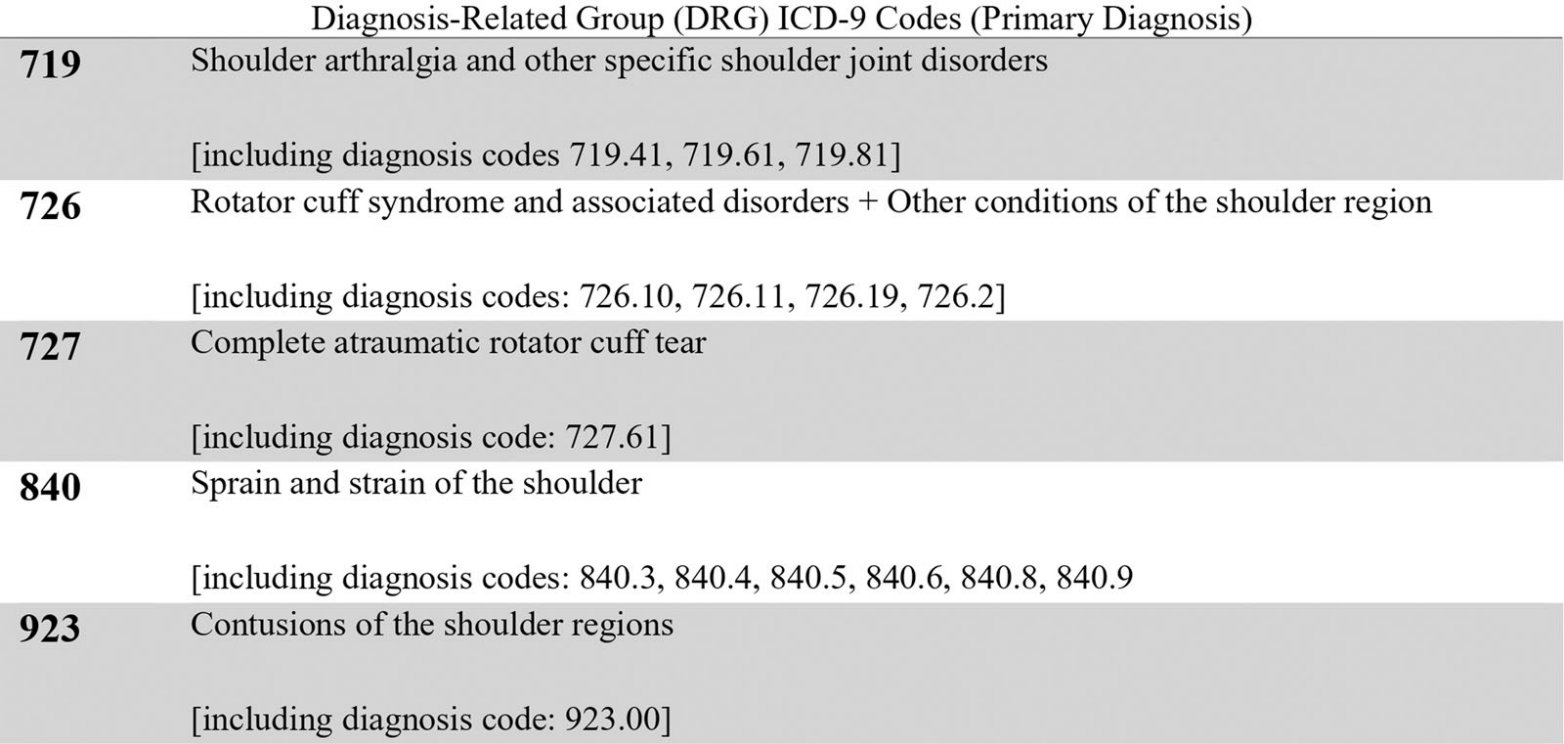
Requested ICD‐9 extraction codes from the SDO database of the Italian Ministry of Health focused on Hospital Admissions with a Primary Diagnosis related to RC Pathology. ICD‐9, International Classification of Diseases, 9th Revision, Clinical Modification; RC, rotator cuff; SDO, Italian national outpatient visits records, national hospital discharge records.

A retrospective analysis was conducted using the SDOs provided by the Italian Ministry of Health for the period 2017 to 2023. Data extraction focused on hospital admissions with a primary diagnosis related to RC disorders, identified using International Classification of Diseases, 9th Revision, Clinical Modification (ICD‐9‐CM) codes. The exact ICD‐9‐CM codes requested can be found in Figure 2 and they were used to identify relevant diagnoses.

**Figure 3:**
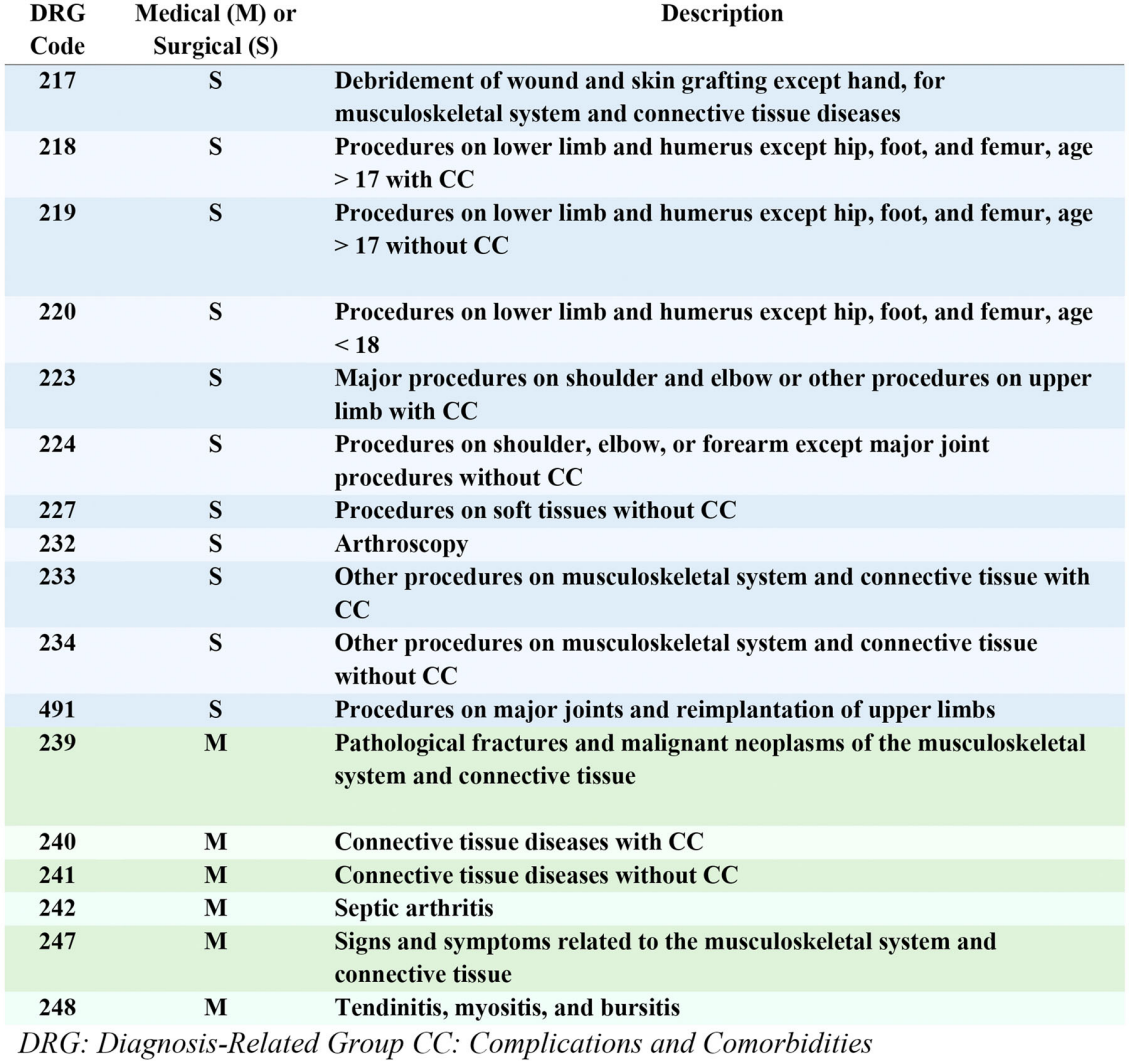
Requested ICD‐9 surgical and medical DRG extraction codes from the SDO database of the Italian Ministry of Health focused on Hospital Admissions with a Primary Diagnosis related to RC Pathology. ICD‐9, International Classification of Diseases, 9th Revision, Clinical Modification; RC, rotator cuff; SDO, Italian national outpatient visits records, national hospital discharge records.

Hospitalisation records were extracted from the national SDO database, which includes information on patient demographics (age, sex); admission and discharge year; diagnosis and procedure codes (primary and secondary); type of admission (elective vs. emergency); type of discharge (e.g., home, transferred, deceased); diagnosis‐related group (DRG) and associated reimbursement. The DRG codes associated to the hospital admission can be stratified into medical and surgical, these are reported in Figure 3. The DRG‐based reimbursement system was used as the primary cost proxy, which explicitly bundles all surgical expenses, including operating theatre use, surgical staff and medical devices, into the total hospitalisation cost.

Only acute care hospitalisations were considered, excluding long‐term care or rehabilitation stays. Cases were included only if the RC disorder was listed as the primary diagnosis, to ensure that hospitalisations were directly attributable to this clinical condition. Data were aggregated and analysed to identify trends in hospitalisation rates and surgical procedures performed, where available.

### INAIL cost analysis

The data analysed refer to the 5‐year period 2019–2023 and processed within INAIL's statistical archives, which feed into the ‘Statistical Database’ and ‘Open Data’, both available online on the official website under the section ‘Activities and Services/Data and Statistics’.

**Figure 4:**
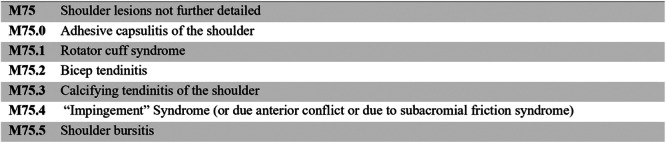
Requested ICD‐10 Extraction Codes from INAIL Database Related to RC Pathology. ICD‐10, International Classification of Diseases, 10th Revision, Clinical Modification Codes; INAIL, Italian National Institute for Insurance against Accidents at Work; RC, rotator cuff.

Procedures were selected based on International Classification of Diseases, 10th Revision, Clinical Modification (ICD‐10‐CM) codes related to RC pathology (M75 and subcategories) focusing exclusively on recognised cases of occupational disease. Exact codes can be seen in Figure 4. Cases were identified as work‐related shoulder injuries.

The most affected economic sectors were identified, along with causal triggering factors including physiological factors and physical agents. Characteristics of affected workers were also extracted, including average age at claim and place of residence.

An average cost per occupational disease case was estimated, encompassing two main components: economic benefits such as daily allowances for temporary total disability, lump‐sum compensation, and direct annuities for impairment of physical and psychological integrity (biological damage), survivor pensions and other expenses, including conservative and surgical healthcare services, personnel costs of the Institute, and administrative costs related to managing injury claims.

The estimated average cost for the period from July 1, 2024, to June 30, 2025, was calculated. This timeframe reflects the annual adjustment of benefits according to Ministerial Decrees effective from July 1st of each year, ensuring periodic updates of economic values underlying the cost estimation. Statistical data from 2019 to 2023 were used to define average parameters.

### Statistical analysis

Descriptive statistical analyses were performed. Frequencies and percentages were used for categorical variables, and mean values with corresponding ranges were used for continuous variables. Data were aggregated and analysed to summarise annual service volumes, costs and trends over the study period (2017–2023 and 2019–2023). Incidence and cost estimates were derived using national administrative data sources, including the Italian Ministry of Health outpatient specialist care database, SDO records and INAIL statistical archives. Attribution percentages were applied to outpatient service categories based on epidemiological and clinical evidence from published studies, to conservatively estimate the proportion of services attributable to RC disorders. Hospitalisation data were grouped by DRG codes, and theoretical remuneration was calculated using national tariff rates defined by the Unified Commission on the Integrated Tariff (CUTI). Average and total costs were computed for each year, stratified by service type, payer (SSN vs. OOP), and facility ownership (public vs. private). Temporal trends and distributions were visually assessed through tabular summaries and graphical representations. All statistical computations and data visualisations were conducted using Microsoft Excel (version 365, Microsoft Corporation).

## RESULTS

### Outpatient cost analysis

#### Average outpatient services costs

The most common outpatient services and their average SSN and OOP outpatient service costs are reported in Table 1.

**Table 1:** Average outpatient service costs: SSN costs and OOP costs.

Service	SSN (€)	OOP (€)
Orthopaedic outpatient visits	22–35	90–150
Shoulder x‐ray	20–35	50–90
Shoulder CT	70–110	120–180
Arthro‐CT	110–160	180–250
Shoulder MRI without CM (soft tissue)	90–130	150–250
Shoulder MRI with CM (vascular, preoperative)	140–180	250–400
Musculo‐tendinous ultrasound	40–70	70–120
Echography‐guided intraarticular infiltration	45–80	90–150
Physiotherapy cycle (10 visits)	120–200	300–600 (30–60€/visit)
Drugs (1 month of continuous therapy)	10–25 (ticket)	30–60 (without reimbursement)

Abbreviations: CM, contrast medium; CT, computed tomography; OOP, out‐of‐pocket costs; SSN, Italian National Health Service (Servizio Sanitario Nazionale).

#### Estimated cost of a conservative therapeutic approach

A standard conservative therapeutic approach often includes the following medical services based on literature reporting and expert opinion [13, 20]:

− 1–2 orthopaedic outpatient visits
− 1 shoulder ultrasound + 1 shoulder x‐ray
− 1‐2 intra‐articular injections
− 10 physiotherapy sessions
− Pharmacological therapy for 1–2 months

Based on estimated costs and the medical services involved in a conservative therapeutic approach, the average cost per patient is estimated at €300–400 for the SSN and €800–1200 in OOP expenses. On a national scale, assuming an active prevalence of 4 million symptomatic patients per year [16, 25, 27], the average expenditure by the SSN to cover all conservative therapy services would amount to: 4,000,000 × €300 ≈ €1.2 billion. Whereas the average OOP expenditure would be 4,000,000 × €800 ≈ €3.2 billion.

#### SSN, OOP and total expenditure based on administrative flow data

Estimated attribution percentages are reported in Table 2. Between 2017 and 2023, a total of 12.911.741 RC pathology‐related services were delivered (Table 3).

**Table 2:** Outpatient services and corresponding attribution percentages.

Service	Estimated attribution %	References
CT Arthrography (shoulder/elbow/knee)	40	[5, 7, 10]
Musculoskeletal ultrasound (Per individual joint or muscle region. Including possible colour Doppler)	45	[1, 3, 13, 18, 21, 30]
Injection of other therapeutic substances (Included: subcutaneous, intramuscular, intravenous administration. Excluded: steroids, hormonal substances, chemotherapeutic agents and biologic drugs)	35	[14, 15, 17]
Initial consultation in physical and rehabilitation medicine	30	[11, 23]
Initial orthopaedic consultation	30	[6, 23, 26]
Shoulder MRI with/without contrast agent (Included: Soft tissues and vascular structures.)	90	[2, 3, 16, 21, 30]
Shoulder x‐ray	80	[18, 21]
Follow‐up orthopaedic consultation	30	[23]

Abbreviations: CT, computed tomography; MRI, magnetic resonance imaging.

**Table 3:** Yearly volume, from 2017 to 2023, of total and rotator cuff (RC) pathology‐related outpatient specialist services calculated using attribution percentages.

Service	2017	2017 RC	2018	2018 RC	2019	2019 RC	2020	2020 RC	2021	2021 RC	2022	2022 RC	2023	2023 RC	Total RC
CT Arthrography (shoulder/elbow/knee)	1.168	467	987	395	882	353	677	271	724	290	671	268	738	295	2339
Musculoskeletal ultrasound	661.329	297.598	660.703	297.316	660.425	297.191	494.283	222.427	600.631	270.284	594.663	267.598	596.089	268.240	1.920.654
Injection of other therapeutic substances	478.423	167.448	484.471	169.565	481.583	168.554	280.097	98.034	315.778	110.522	337.750	118.213	341.838	119.643	951.979
Initial consultation in physical and rehabilitation medicine	781.773	234.532	775.123	232.537	774.268	232.280	533.593	160.078	708.589	212.577	754.288	226.286	818.351	245.505	1.543.795
Initial orthopaedic consultation	1.240.799	372.240	1.235.027	370.508	1.262.389	378.717	889.870	266.961	1.167.546	350.264	1.320.416	396.125	1.437.870	431.361	2.566.176
Shoulder MRI with/without contrast agent	61.204	55.084	64.007	57.606	64.292	57.863	39.729	35.756	48.946	44.051	49.210	44.289	43.608	39.247	333.896
Shoulder x‐ray	655.052	524.042	651.213	520.970	635.174	508.139	461.430	369.144	540.062	432.050	546.860	437.488	553.731	442.985	3.234.818
Follow‐up orthopaedic consultation	1.231.215	369.365	1.221.072	366.322	1.230.899	369.270	848.123	254.437	1.032.899	309.870	1.126.630	337.989	1.169.441	350.832	2.358.085
**Total**	**5.110.963**	**2.020.775**	**5.092.603**	**2.015.219**	**5.109.912**	**2.012.367**	**3.547.802**	**1.407.108**	**4.415.175**	**1.729.907**	**4.730.488**	**1.828.256**	**4.961.666**	**1.898.109**	**12.911.741**

Abbreviations: CT, computed tomography; MRI, magnetic resonance imaging.

The annual expenditure related to RC pathology for the year 2023 reached approximately €1.7 billion, details are reported in Table 4.

**Table 4:** Total service volumes, SSN and OOP costs, and RC pathology‐related service volumes, SNN, OOP and total expenditure, for each service for the year of 2023.

Service	2023 service volumes (Total)	SSN expenditure (Total)		OOP expenditure (Total)	2023 service volumes (RC)	2023 SSN expenditure (RC)	2023 OOP expenditure (RC)	Total expenditure (RC)	RC expenditure % SSN	RC expenditure % OOP
CT Arthrography (shoulder/elbow/knee)	738	6.269.266 €	670.759 €		295	2.507.706 €	268.304 €	2.776.010 €	90	10
Musculoskeletal ultrasound	596.089	415.722.308 €	353.449.794 €		268.240	187.075.039 €	159.052.407 €	346.127.446 €	54	46
Injection of other therapeutic substances	341.838	261.159.325 €	217.644.538 €		119.643	91.405.764 €	76.175.588 €	167.581.352 €	55	45
Initial consultation in physical and rehabilitation medicine	818.351	78.977.399 €	92.771.081 €		245.505	23.693.220 €	27.831.324 €	51.524.544 €	46	54
Initial orthopaedic consultation	1.437.870	143.536.678 €	338.267.970 €		431.361	43.061.003 €	101.480.391 €	144.541.394 €	30	70
Shoulder MRI with/without contrast agent	43.608	295.699.689 €	40.317.389 €		39.247	266.129.720 €	36.285.650 €	302.415.370 €	88	12
Shoulder x‐ray	553.731	267.169.000 €	354.526.673 €		442.985	213.735.200 €	283.621.338 €	497.356.538 €	43	57
Follow‐up orthopaedic consultation	1.169.441	153.672.960 €	436.895.754 €		350.832	46.101.888 €	131.068.726 €	177.170.614 €	26	74
Total	4.961.666	1.622.206.625 €	1.834.543.958 €		1.898.109	873.709.540 €	815.783.729 €	1.689.493.269 €	52	48

Abbreviations: CT, computed tomography; MRI, magnetic resonance imaging; OOP, out‐of‐pocket costs; SSN, Italian National Health Service (Servizio Sanitario Nazionale).

### Hospital discharge records (Schede di Dimissione Ospedaliera, SDO) cost analysis (2017–2023)

#### Hospital admissions for RC pathology

**Figure 5:**
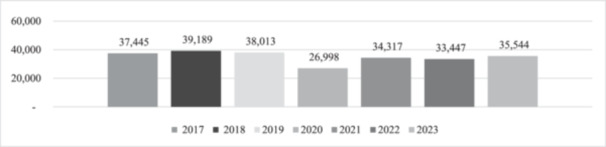
Hospital admissions with primary diagnosis of RC pathology (2017–2023). RC, rotator cuff.

The analysis of SDO based solely on the primary diagnosis recorded approximately 244,953 discharges between 2017 and 2023. Average hospitalisations per year are reported in Figure 5.

**Figure 6:**
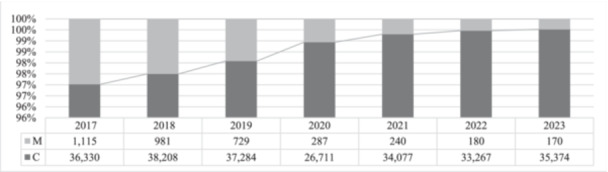
Hospital admissions for RC pathology under medical (M) versus surgical (S) DRGs (2017–2023). DRG, diagnosis‐related group; RC, rotator cuff.

On average, 98% of admissions were made under surgical DRGs, the trend in medical versus surgical admission codes over a 7‐year period are depicted in Figure 6.

#### Hospital admissions under surgical DRG codes for RC pathology

**Figure 7:**
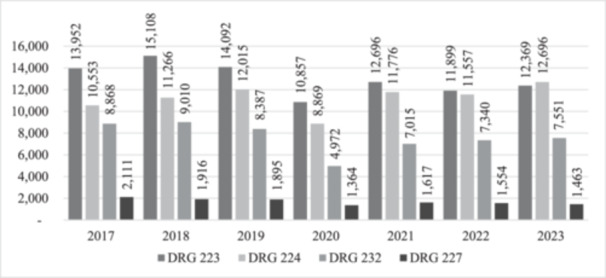
Most used surgical DRG codes for RC pathology‐related hospital admissions. DRG, diagnosis‐related group; RC, rotator cuff.

As seen in Figure 7, more than 90% of surgical DRG hospital admissions for RC pathology fall into DRG codes 223, 224 and 232. There is also an increase in admissions under DRG 224 and 232 (the latter being at high risk of inappropriateness).

95% of the primary surgical procedures associated with these diagnoses are RC repairs or other shoulder soft tissue repairs. Only about 2% of hospitalisations with a primary diagnosis of shoulder and RC pathology result in a shoulder arthroplasty.

#### Hospital admissions for RC pathology in private versus SSN facilities

Over 70% of hospital admissions were carried out in accredited private facilities, with only 1% occurring in nonaccredited private centres (not affiliated with the SSN). Over 7 years, public healthcare facilities have lost 13% of admissions for RC pathology patients to the benefit of the private sector.

#### Age and sex stratification of hospital discharge records for RC and shoulder pathology

**Figure 8:**
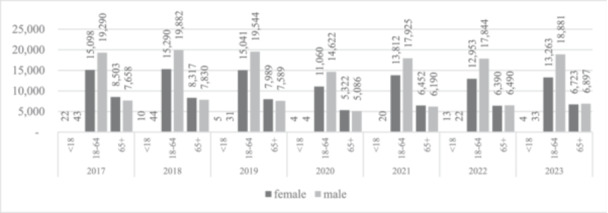
Hospital discharge records for RC and shoulder pathology according to patient age and sex. RC, rotator cuff.

While total hospital discharges from 2017 to 2023 were highest in the 18–64 age group, the volume of patients aged >65 was disproportionately high. Despite representing a smaller segment of the general population, the >65 group consistently accounted for a total number of discharges that was approximately half of the volume seen in the entire 18–64 cohort. Visual representation of these findings can be seen in Figure 8.

#### Theoretical remuneration according to DRG codes

**Figure 9:**
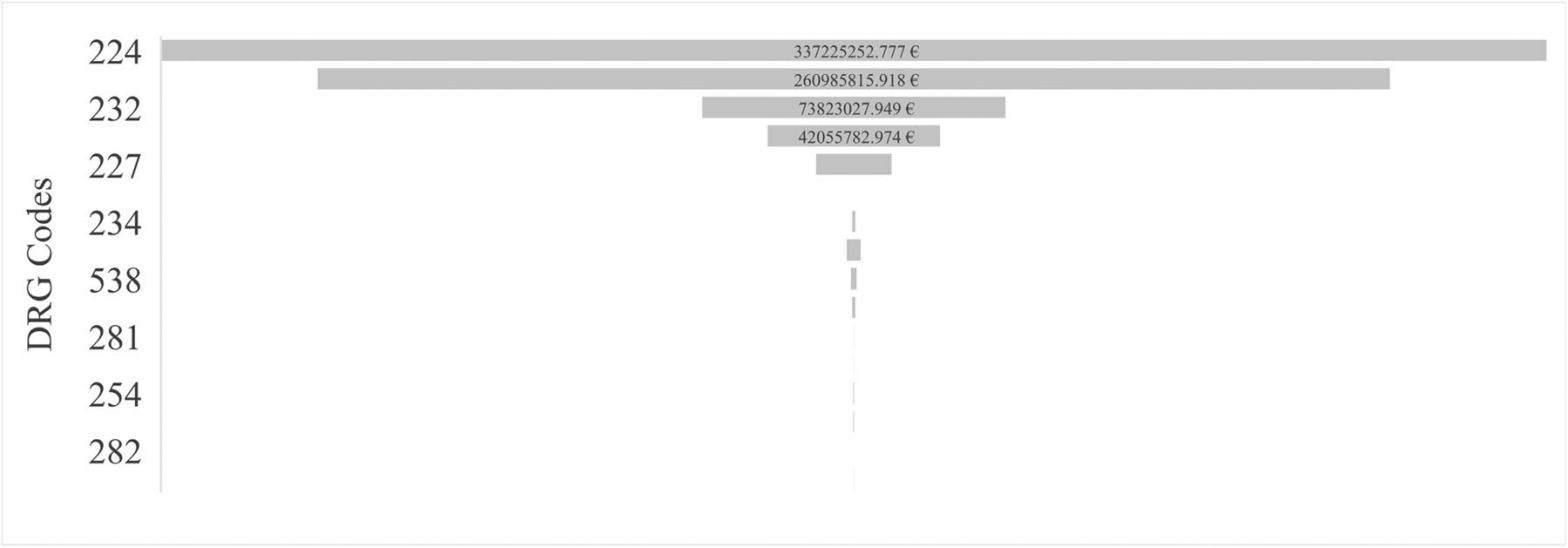
Tornado diagram showing the impact of theoretical remuneration on total costs by DRG for shoulder and RC pathologies from 2017 to 2023. DRG, diagnosis‐related group; RC, rotator cuff.

**Figure 10:**
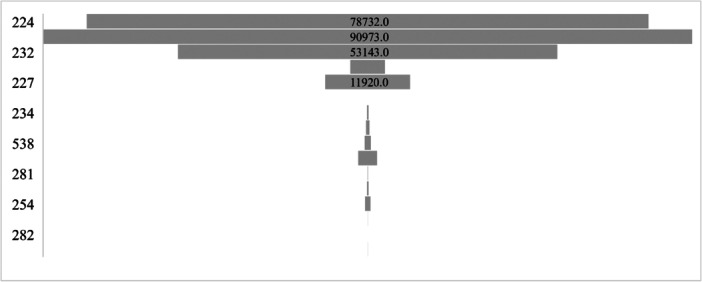
Tornado diagram showing the impact of discharge volumes of DRG for shoulder and RC pathologies from 2017 to 2023. DRG, diagnosis‐related group; RC, rotator cuff.

The Hospitalisation Tariff Nomenclature, defined by the CUTI, which standardises costs for inpatient services provided by accredited public and private healthcare facilities was used for cost estimates reported in Table 5 and Figures 9 and 10.

**Table 5:** Annual trends in hospital discharges and theoretical remuneration by DRG for shoulder and RC pathologies (2017–2023).

DRG Code	n° 2017	2017 TV	n° 2018	2018 TV	n° 2019	2019 TV	2020	2020 TV	2021	2021 TV	2022	2022 TV	2023	2023 TV	2017‐2023	TV 2017‐2023
217	8	43,038	14	61,528	30	230,334	48	368,535	125	959,727	109	786,324	156	972,529	**490**	**3,422,018**
219	0	0	0	0	0	0	0	0	0	0	5	9437	0	0	**5**	**9437**
223	13,952	40,909,402	15,108	43,509,367	14,092	40,477,972	10,857	30,959,052	12,696	35,932,474	11,899	33,658,190	12,369	35,539,356	**90,973**	**260,985,816**
224	10,553	45,611,940	11,266	48,731,661	12,015	51,348,283	8869	38,046,256	11,776	50,371,628	11,557	49,188,627	12,696	53,926,855	**78,732**	**337,225,253**
226	0	0	0	0	0	0	0	0	0	0	0	0	0	0	**0**	**0**
227	2111	3,268,959	1916	2,961,893	1895	2,927,622	1364	2,106,053	1617	2,499,462	1554	2,399,436	1463	2,260,163	**11,920**	**18,423,592**
232	8868	12,280,577	9010	12,455,828	8387	11,639,602	4972	6,920,223	7015	9,758,716	7340	10,235,477	7551	10,532,602	**53,143**	**73,823,028**
234	36	133,978	42	180,414	33	131,756	32	101,466	25	97,058	20	92,577	25	78,395	**213**	**815,648**
247	90	63,671	11	2353	46	9843	27	5777	0	0	8	1711	33	13,187	**215**	**96,545**
248	743	245,853	816	237,352	595	140,912	177	59,701	138	49,953	107	38,571	109	43,941	**2685**	**816,286**
254	230	84,841	154	34,533	88	17,921	83	20,698	102	30,557	65	21,876	28	216,489,519	**750**	**232,077**
281	52	46,826	0	0	0	0	0	0	0	0	0	0	0	0	**52**	**46,826**
282	0	0	0	0	0	0	0	0	0	0	0	0	0	0	**0**	**0**
481^a^	0	0	0	0	4	239,224	0	0	0	0	0	0	0	0	**4**	**239,224**
491	546	4,676,671	697	5,970,036	712	6,098,516	482	4,128,490	707	6,055,690	713	6,107,082	1053	901,929,521	**4910**	**42,055,783**
538	256	427,912	155	260,666	116	195,971	87	148,640	116	19,629,292	70	116,613	61	103,044,653	**861**	**1,449,141**
Total	37,445	107,762,962	39,189	114,374,924	38,013	113,227,627	26,998	82,496,359	34,317	104,991,834	33,447	101,903,501	35,544	111,689,494	244,953	**739,640,676 €**

*Note*: Bold indicate total values from 2017‐2023 of number of discharges and their corresponding total tariff value in euros.

Abbreviations: DRG, diagnosis‐related group; n°: number of hospital discharges; RC, rotator cuff; TV: tariff value in euros.

a Note that DRG code 481 corresponds to ‘bone marrow transplantation’, which was likely erroneously associated to shoulder and RC pathology–related hospital admissions.

Table 5 presents the yearly number of hospital discharges for primary diagnoses related to shoulder and RC pathologies, grouped by DRG code, along with their corresponding theoretical remuneration. The data reflect trends in clinical activity and provide an estimate of the potential economic burden on the SSN from 2017 to 2023 which amounts to over €739 million.

Figures 9 and 10 show that DRG codes 224, 223 and 232 account for both the highest patient volumes and the largest total costs. Notably, DRG code 491, representing arthroplasty procedures, involves a relatively small patient population, approximately 5000 cases, but incurs very high costs, totalling over 42 million euros.

Additionally, DRG code 227, characterised by a vague clinical description, presents a potential risk of inappropriate use, but nonetheless ranks among the top five DRG codes in terms of total spending.

### Italian national INAIL cost analysis

#### Positively confirmed cases of occupational shoulder injuries and corresponding demographics

Musculoskeletal disorders are the most frequently reported occupational diseases, in 2023, they accounted for 70% of all reports, totalling over 51,000 claims, mainly consisting of ‘soft tissue disorders’. More than 24,000 of those claims primarily involved the upper limb including shoulder and elbow pathologies.

Shoulder injury‐related occupational diseases reported in 2023 amounted to 16,000 cases, while for the most recent 5‐year reporting period available (2019–2023), the total number of claims exceeded 64,000 cases. However, not every claim is accepted, as each one undergoes an assessment process to determine whether it qualifies for protection and compensation.

Table 6 reports the positively confirmed cases of shoulder injury recognised by the institute from 2019 to 2023, identified using the ICD‐10 code ‘M75’ and its subcategories, marked with the administrative classification ‘P—positive’. Data are reported in Table 6.

**Table 6:** Positively confirmed cases of occupational shoulder injuries by type of disease and year of registration; total for 2019–2023.

Type of pathology (ICD‐10 code)	Year of registration					Total 5‐year period
	2019	2020	2021	2022	2023	
M75: Shoulder lesions not further detailed	2.063	1.324	1.706	1.811	1.890	8.794
M75.0: Adhesive capsulitis of the shoulder	6	3	‐	4	1	14
M75.1: Rotator cuff syndrome	4.288	3.446	4.156	4.473	4.508	20.871
M75.2: Bicep tendinitis	120	80	104	115	119	538
M75.3: Calcifying tendinitis of the shoulder	464	378	399	406	349	1.996
M75.4: ‘Impingement’ syndrome	449	355	331	294	240	1.669
M75.5: Shoulder bursitis	42	51	56	66	84	299
Total confirmed cases	**7.432**	**5.637**	**6.752**	**7.169**	**7.191**	**34.181**

Abbreviation: ICD‐10, International Classification of Diseases, 10th Revision, Clinical Modification Codes.

The percentage of cases of injury and corresponding economic sectors of affected workers can be seen in Table 7.

**Table 7:** Positively confirmed cases of occupational shoulder injuries by economic activity (2019–2023).

Economic activity (INAIL management/ATECO‐ISTAT classification)	N°	%
Agriculture	9.035	26
Industry and services, and public sector	**25.146**	**74**
of which, mainly,		
F—Construction	6.681	20
C—Manufacturing sector	5.272	15
G—Commerce	1.999	6
S—Other service activities	1.462	4
Q—Health and social assistance	1.148	3
Total	**34.181**	**100**

Abbreviations: ATECO‐ISTAT, Economic Activities‐Italian National Institute of Statistics; INAIL, Italian National Institute for Insurance against Accidents at Work.

As reported in Table 8 RC pathologies are almost exclusively attributable to physiological factors, particularly repetitive work. Only a small portion is associated with exposure to physical agents.

**Table 8:** Positively confirmed cases of occupational shoulder injuries by causal agent (2019–2023).

Causal agent		N°	%
Physiological factors		33.306	97
Of which:	Repetitive labour	21.930	64
	Different movements	4.767	14
	Lifting a load	2.536	7
	Positioning at work	2.388	7
	Transport of loads	412	1
	Fast labour	350	1
	Other	923	3
Physical agents		875	3
Of which:	Friction, rubbing (wear and tear)	525	2
	Vibration	350	1
Total		**34.181**	**100**

According to data reported by INAIL, 72% of recognised cases involve male workers, with an average age at the time of claim of 57 years (58 for men, 56 for women).

#### Reporting on direct costs (INAIL data)–Estimate of the average cost of an occupational shoulder injury

The estimate of the average cost of an occupational disease is based on two main components, economic benefits and administrative costs. Between 2019 and 2023, a total of 34,181 confirmed cases of occupational shoulder diseases were recorded, this total was divided by type of compensation received which is shown in Table 9.

**Table 9:** Confirmed occupational shoulder diseases in the 2019–2023 by type of compensation, data reported in percentage.

No compensation	Temporary allowance	Lump‐sum compensation	Direct annuity	Survivor pension
21.30%	**0.70%**	**57.14%**	**20.86%**	**0.01%**

The average cost per case was calculated using a weighted approach based on the proportion of each compensation type and their associated costs. The overall average cost per occupational shoulder disease case for 2024 was estimated at approximately €25,700.

#### Qualitative overview of indirect costs

Indirect costs primarily stem from reduced work productivity and long‐term disability outcomes documented by INAIL. These include extended periods of work absence covered by the institute, compensation for diminished working capacity, cases of early retirement or occupational reallocation, and broader impacts on individuals' psychological well‐being and overall quality of life.

#### Estimate of total cost

Based on INAIL data, the average cost per case varies depending on clinical severity and the extent of required interventions. Annual total costs for INAIL could amount to several €100 million.

## DISCUSSION

The present analysis was based on national‐level data involving an average population of 59 million residents eligible for SSN care. By integrating outpatient records and hospital discharge data from both public and accredited private facilities, alongside INAIL compensation records, we estimated that the annual economic burden of RC pathology exceeds €2 billion. To put this in perspective, this figure represents approximately 1.5% of the annual Italian National Health Fund, which averaged roughly €125 billion per year during the study period.

The present analysis was based on outpatient visits records when considering a standard conservative therapeutic approach, data from national hospital discharge records, and finally data from INAIL on work‐related injuries and their compensation. The results complied suggest that the economic burden of RC pathology in Italy, exceeds €2 billion per year. This estimate remains conservative and future cost‐analysis would benefit especially from consideration of indirect costs and physiotherapy‐related outpatient services within conservative treatment regimens and in the postoperative phase.

In terms of estimated outpatient costs related to an initial conservative approach, data reveal over 12 million services being delivered over a 7‐year period, with almost €1.7 billion spent in 2023 alone. Slightly less than half of the total share of this yearly cost was covered via OOP spending, while the rest is represented by SSN expenditure. This figure is consistent with high individual‐spending.

Unfortunately, a dual mode of access and funding introduces critical elements of inefficiency and inequality, particularly when services are not included in a structured diagnostic and treatment pathway, are repeated multiple times without significant clinical impact, or even replace definitive interventions with excessive use of palliative treatments. Within this context, there is not only potential for waste of both public and private resources, but also delays in access to appropriate surgical care, leading to chronic progression of the condition, a subsequent decline in patient quality of life and increased future costs for the system [13, 22].

A study evaluating the cost‐effectiveness of surgical treatment for full‐thickness RC tears found that operative repair, after an initial trial of conservative management, leads to significant lifetime societal savings, reaching billions of dollars in the United States annually [15]. The study showed that, particularly in patients under the age of 61, the reduction in indirect costs outweighed the additional direct costs of surgery [15]. Across all age groups, RC repair was more cost‐effective and provided greater health utility than nonoperative treatment, supporting payer coverage for surgery following failed conservative care [15]. Such conclusions are not stand‐alone in the rationale that surgery is cost‐effective after an initial trail of conservative therapy [13, 22, 26].

Other evidence suggests that increasing the volume of healthcare does not inherently lead to better results, neither the number of medical visits nor the total financial costs of conservative care were significantly associated with improvements in shoulder pain and disability at 1 year [2]. Instead, literature seems to favour following the quality and response to therapy as opposed to its quantity. Additionally, the implementation of standardised, evidence‐based clinical practice guidelines has been shown to successfully shift management toward more appropriate treatment and the prevention of unnecessary surgeries [2, 25]. For example, in the Netherlands, the introduction of a multidisciplinary guideline led to a 10% decrease in ‘impingement‘ diagnoses and a significant reduction in surgical intervention [25]. Specifically, the percentage of patients with RC tears treated surgically dropped from 30% to 25% following the guideline's dissemination [25].

Overall, yearly outpatient costs could likely be reduced and better controlled with the introduction of more structured care pathways and rehabilitation protocols as well as timely access to surgery, only when truly indicated, all of which should be applied to both private and public sectors. Additionally, these protocols, although cost‐effective, could work to improve patient outcomes thanks to a stronger focus on quality of care and patient‐response.

Hospital admissions also contribute to generating high public and private expenditure, the 7 years analysed saw steady patient volumes, up until 2020, where the COVID‐19 pandemic led to decline in admissions. Numbers rapidly rose again, and as of 2023 had almost normalised to prepandemic figures. Over the same span of time, hospital admissions under medical DRG codes related to RC pathology are decreasing, in favour of surgical codes involving RC repair, and more vague shoulder interventions such as ‘soft tissue repairs’, that likely, at least in part, are related to RC pathologies.

Trends in hospital admissions also revealed that accredited private facilities saw a significant increase in number, representing 70% or more of total admissions over the last 5 years, emphasising a significant and continuous loss of volume for SSN facilities. Here we can see once again a dual mode of access, which as discussed has the potential to lead to economic losses and may negatively burden individuals sustaining OOP costs.

Overall, hospital admissions resulted in over €739 million in theoretical remuneration over 7 years, with shoulder arthroplasty representing only 2% of patient volume, but still contributing much more in costs compared to other procedures. This datapoint further sustains the importance of avoiding chronic progression of RC pathology, that may result in arthroplasty, by prioritising timely surgical intervention.

Another important portion of national expenditure is attributable to work‐related injuries. RC pathology incidence is particularly high in labourers who perform biomechanical overloading due to repetitive and strenuous work‐related activity. This is related to the extrinsic pathway of RC pathogenesis, where repetitive overhead activities and biomechanical overloading can lead to mechanical compression and physical abrasion of the tendons within the subacromial space, eventually leading to a progressive degradation of tendon tissue [24]. In fact, between 2019 and 2023, over 30 thousand confirmed cases of occupational shoulder injuries were recorded. In the year 2024, each of these patients would've been granted, on average, €25,700 per injury, likely totalling a national expenditure of several €100 million annually.

It is clear from this data that RC pathology generates a significant economic impact for the INAIL system, contributing significantly to national yearly expenditure. The high costs once again justify the effort towards prevention, early diagnosis and the optimisation of therapeutic pathways of this pathology to reduce public cost while simultaneously improving clinical outcomes for injured workers.

The present study has several limitations, most notably those related to the use of administrative data derived from generic or potentially inaccurate procedural and diagnostic codes. These limitations made it challenging to reliably distinguish shoulder‐related pathology from RC–specific disease. Furthermore, inconsistencies in coding practices were identified given that procedural codes were not consistently linked to corresponding diagnostic codes. As a result, estimated attribution percentages were required to approximate the proportion of procedures specifically attributable to RC pathology. This approach inevitably introduced a degree of uncertainty within the results. These findings also highlight the need for improved accuracy, education and standardisation in the use of administrative coding systems by healthcare professionals, which is critical not only for economic analyses but also for large‐scale epidemiological research on surgical interventions.

It is also important to acknowledge the inherent ‘data gap’ regarding outpatient and diagnostic services performed within nonaccredited private practices. In the Italian system, these services do not generate administrative records within the Ministry of Health's databases, essentially ‘escaping’ national tracking. While the exact volume of these services is difficult to quantify precisely due to the lack of a centralised registry, evidence suggests that a significant portion of specialised orthopaedic consultations and initial diagnostic imaging is sought privately to bypass public waiting lists, or due to other patient‐driven necessities or preferences. Consequently, the €1.7 billion estimated for outpatient costs must be interpreted as a conservative, lower‐bound figure. While this missing data does not undermine the reliability of the costs captured from the SSN and accredited‐private providers, it implies that the true economic burden of RC pathology, particularly the OOP component, is likely higher than reported in the present analysis.

It should also be noted that costs related to GP consultations were not included in this analysis. In the Italian SSN, GPs serve as the primary and initial approach to diagnosis and treatment of RC pathology. However, because GPs are reimbursed primarily through capitation rather than the specialist fee‐for‐service nomenclature tracked in the national outpatient database, these primary care costs are not captured in our data. This represents another factor contributing to the conservative nature of our total cost estimate.

The use of approximation of various variables was further adopted due to the unavailability of complete therapeutic flux due to the division between private and public sectors, the former for which data was not retrievable.

Although these methods are not optimal, conservative underestimating approximations were employed to minimise bias and to ensure that results reflected true values as closely as possible, informed by high‐quality evidence‐based sources.

Another inconsistency was that outpatient and hospital discharge record cost‐analysis was carried out on data from 2017 to 2023, whereas INAIL cost data was only accessible from 2019 onwards due to a system change that prevented retrieval of more recent records. Despite this limitation, the analysis remains valid as INAIL costs represents a more stable component of the overall economic burden, with perhaps more limited year‐to‐year variability compared to healthcare utilisation data. While inclusion of earlier outpatient and hospital discharge information, including those prepandemic figures, ensures that longer‐term trends in clinical management and healthcare resource use are accurately captured, thereby preserving the robustness and representativeness of the cost‐of‐illness estimates.

Finally, physiotherapy and rehabilitative medicine‐related costs were not evaluated. Given the extensive analysis made it goes beyond the scope of the present study, however it is something worth exploring in future studies. The lack of data gathered on this aspect further contributes to the underestimation of total comprehensive costs related to RC pathology given the large role of physiotherapy visits and treatment have in both the conservative treatment approach and the postoperative period.

## CONCLUSION

The annual economic burden of RC pathology in Italy exceeds €2 billion euros yearly, coming from both private and public spending related to conservative outpatient treatment services, hospital admissions and expenditure for work‐related injuries. This figure provides a lower‐bound estimate of the total economic burden, and future cost‐analysis would benefit especially from consideration of indirect costs and physiotherapy‐related outpatient services within conservative treatment regimens and in the postoperative phase. Finally, these findings support the need for nationally standardised diagnostic and therapeutic pathways.

## AUTHOR CONTRIBUTIONS

**Umile Giuseppe Longo**: Conceptualisation; formal analysis; methodology; writing—review and editing. **Martina Marino**: Formal analysis; methodology; original draft preparation; writing—review and editing. **Vincenzo Candela**: Formal analysis; methodology; writing—review and editing. **Silvia D'Amario**: Investigation; data curation; methodology; writing—review and editing. **Daniela Martini**: Investigation; data curation; methodology; writing—review and editing. **Andrea Bucciarelli**: Investigation; data curation; methodology; writing—review and editing. **Ranieri Poli**: Investigation; data curation; methodology; writing—review and editing. **Pieter D'Hooghe**: Methodology; writing—review and editing. **Alessandro de Sire**: Methodology; writing—review and editing. **Michele Mercurio**: Methodology; writing—review and editing. **Arianna Carnevale**: Formal analysis; methodology; writing—review and editing.

## CONFLICT OF INTEREST STATEMENT

The authors declare no conflicts of interest.

## ETHICS STATEMENT

The IRB of Campus Bio‐Medico University of Rome ruled that no formal ethics approval was required in this particular case. Access to the database is on request. All methods were carried out in accordance with relevant guidelines and regulations.

## Data Availability

All relevant data analysed in this study is included in this published article and its supplementary information files. Further inquiries can be directed to the corresponding author.
